# Neoadjuvant therapy combined with immunotherapy for anal sphincter preservation rate, clinical efficacy, and safety in rectal cancer patients: a meta-analysis

**DOI:** 10.3389/fonc.2026.1866026

**Published:** 2026-08-07

**Authors:** Sijie Chen, Yuansha Ge, Xue He, Wenkang Zhang, Jie Li

**Affiliations:** 1 Guang’anmen Hospital, China Academy of Chinese Medical Sciences, Beijing, China; 2 Department of Integrated Chinese and Western Medicine, Zhejiang Cancer Hospital, Hangzhou Institute of Medicine (HIM), Chinese Academy of Sciences, Hangzhou, Zhejiang, China

**Keywords:** anal sphincter preservation, clinical efficacy, immunotherapy, meta-analysis, neoadjuvant therapy, rectal cancer

## Abstract

**Background:**

Neoadjuvant therapy combined with immunotherapy has emerged as a promising strategy for rectal cancer (RC), but its efficacy in anal sphincter preservation, clinical outcomes, and safety profile remains to be systematically validated. This meta-analysis aims to comprehensively evaluate the anal sphincter preservation rate, clinical efficacy, and safety of neoadjuvant therapy combined with immunotherapy in rectal cancer patients, providing evidence-based support for clinical decision-making.

**Objective:**

To assess the anal sphincter preservation rate, key clinical efficacy indicators (including pathological complete response rate, R0 resection rate, and tumor regression grade), and safety indicators (adverse events and postoperative complications) of neoadjuvant therapy combined with immunotherapy in rectal cancer patients. We systematically synthesize the absolute sphincter-preservation, pathological-response, and safety proportions of neoadjuvant immunotherapy in rectal cancer patients, with any comparison to historical data of conventional regimens presented as indirect and exploratory only.

**Methods:**

This study was prospectively registered in PROSPERO (registration number: CRD420251159170) and reported in accordance with the Preferred Reporting Items for Systematic Reviews and Meta-Analyses (PRISMA) guidelines. The methodological quality of this systematic review was evaluated using the AMSTAR 2 tool. A comprehensive electronic search was performed across eight databases (PubMed, Embase, Cochrane Library, Web of Science, Sinomed, CNKI, WanFang, and VIP) up to February 2, 2026. Eligible studies included randomized controlled trials (RCTs), single-arm trials, and observational studies (cohort studies, case-control studies) investigating neoadjuvant therapy combined with immunotherapy for rectal cancer. Data extraction was independently performed by two authors, and risk of bias was assessed using the ROBINS-I tool (for non-randomized studies) and the Cochrane Collaboration risk of bias tool (for RCTs). The GRADE approach was used to evaluate the certainty of evidence. Statistical analysis was conducted with R Studio 4.3.2 software, employing random-effects or fixed-effects models based on heterogeneity (assessed by I² statistic and chi-squared test). This meta-analysis does not include controlled comparative trials and that all analyses are single-arm proportion pooling without direct between-group comparisons.

**Results:**

A total of 16 studies involving 540 rectal cancer patients were included. The pooled anal sphincter preservation rate was 0.90 (95% CI: 0.87–0.93) with moderate heterogeneity (I²=37.4%, P = 0.0716). The R0 resection rate was 0.98 (95% CI: 0.96–1.00) (I²=51.4%, *P* = 0.0361), and the pathological complete response (pCR) rate was 0.34 (95% CI: 0.29–0.38) (I²=71.8%, *P* < 0.0001). The overall complete response (CR) rate was 0.49 (95% CI: 0.44–0.54) (I²=66.0%, *P* = 0.0007), and the tumor regression grade (TRG) 0–1 rate was 0.55 (95% CI: 0.49–0.62) (I²=78.6%, *P* < 0.0001). Regarding safety, the incidence of grade 1–2 adverse events was 0.80 (95% CI: 0.71–0.89) (I²=0.0%, *P* = 0.8159), grade ≥3 adverse events was 0.17 (95% CI: 0.13–0.21) (I²=97.4%, *P* < 0.0001), and postoperative complications rate was 0.24 (95% CI: 0.18–0.30) (I²=92.4%, P<0.0001). Pooled estimates within clinically defined subgroups were generally consistent and are reported descriptively only.

**Conclusions:**

Neoadjuvant therapy combined with immunotherapy was associated with favorable pooled proportions of sphincter preservation and R0 resection in the included studies, although these estimates are derived predominantly from single-arm studies. This regimen warrants further investigation for organ preservation strategies, but confirmatory evidence from high-quality RCTs is needed. Given that the current evidence is predominantly of low-to-moderate certainty, high-quality, large-scale RCTs with long-term follow-up are needed to further validate these findings and optimize treatment regimens.

**Systematic review registration:**

https://www.crd.york.ac.uk/prospero/display_record.php?RecordID=CRD420251159170, identifier CRD420251159170.

## 1 Introduction

Rectal cancer is a major subtype of colorectal cancer — the third most common malignancy and the second leading cause of cancer-related deaths worldwide. In 2022, the global age-standardized incidence rate was 19.7 per 100,000, with approximately 1.93 million new cases and over 900,000 deaths. Notably, the incidence among individuals under 50 years of age continues to rise (1). Approximately one-third of colorectal cancers occur in the rectum, and a large proportion of rectal cancer patients are already diagnosed at locally advanced stages at initial presentation (2).

Anal resection, as one of the core surgical interventions for achieving radical cure in rectal cancer patients, primarily aims to completely remove the tumor, the involved intestinal segment, and surrounding potentially infiltrated tissues, such as the anal sphincter and perianal fat, thereby thoroughly eliminating tumor cells and reducing the risk of local recurrence (3). Although the application of minimally invasive surgical techniques and standardized neoadjuvant therapy has improved the rate of organ-preserving treatment, the rate of anal resection remains relatively high. Particularly, many patients with ultra-low rectal cancer are ultimately scheduled to undergo permanent colostomy. For these patients, abdominoperineal resection (APR) has traditionally been the preferred and recommended surgical approach. However, beyond resulting in a permanent stoma, APR is associated with significant short-term morbidity and mortality (4), as well as perineal wound complications, urogenital dysfunction, chronic pain, and psychological disorders, imposing substantial survival burdens and psychological distress on patients (5, 6). Nevertheless, anal sphincter preservation is not without oncological risks. Some studies have indicated an inherent trade-off between sphincter preservation and local recurrence control when effective neoadjuvant therapy is unavailable. For instance, a study on ultra-low rectal cancer reported that patients with clinically staged T3 tumors who did not receive neoadjuvant therapy and underwent sphincter-preserving intersphincteric resection experienced a 3-year local recurrence rate as high as 21.6% (7). This suggests that in certain high-risk patients, sphincter-preserving surgery may increase local recurrence risk due to insufficient resection margins, presenting challenges for balancing anal function and oncological safety in clinical decision-making.

In clinical practice, neoadjuvant chemoradiotherapy, total mesorectal excision, and adjuvant chemotherapy constitute the standard treatment regimen for locally advanced rectal cancer (8). Furthermore, the concept of neoadjuvant therapy has evolved from solely controlling local recurrence to focusing on enhancing tumor regression grades, achieving organ preservation, and improving long-term survival. Particularly, organ preservation is no longer merely an opportunistic strategy following a favorable treatment response but should be a therapeutic direction actively discussed by physicians and patients early in the treatment process (9). Immune checkpoint inhibitors (ICIs), as an emerging class of oncological therapy, have been actively explored in the neoadjuvant setting for rectal cancer. For MSI-H/dMMR populations, neoadjuvant ICIs have achieved high complete response rates and favorable tolerability; for MSS/pMMR populations, the efficacy of neoadjuvant ICIs combined with chemoradiotherapy is still under investigation, with early single-arm studies showing encouraging preliminary results (10, 11). The improved tumor response rates create opportunities for both sphincter-preserving surgery and “watch and wait” non-surgical management strategies, broadening the therapeutic spectrum for anal function preservation (12).

Despite these advances, the application of ICIs in the neoadjuvant treatment of rectal cancer still faces numerous unresolved questions: How should the optimal treatment regimen be determined? How can the benefiting population be precisely identified? Does its efficacy and safety profile comprehensively surpass that of traditional neoadjuvant chemoradiotherapy? Definitive answers to these critical questions are currently lacking (13). Therefore, we conducted this systematic review and meta-analysis to synthesize the anal sphincter preservation rate, clinical efficacy and safety profile of neoadjuvant therapy combined with immunotherapy in rectal cancer patients, with a particular focus on sphincter preservation outcomes. We further conducted exploratory subgroup analyses to describe pooled estimates within clinically defined subgroups, aiming to provide evidence-based reference for clinical decision-making of neoadjuvant immunotherapy and sphincter preservation strategies.

## 2 Method

### 2.1 Literature search

This study was prospectively registered in PROSPERO(CRD420251159170). The work has been reported in line with the Preferred Reporting Items for Systematic Reviews and Meta-Analyses (PRISMA) guidelines (14). The methodological quality of the systematic review was appraised using the AMSTAR 2 tool. A comprehensive search of eight databases (PubMed, Embase, Cochrane Library, Web of Science, Sinomed, CNKI, WanFang and VIP) was conducted for relevant studies. The date of the last search was 2 February 2026. The following MeSH and free words were used in the searches:”((“rectal neoplasms”[Title/Abstract] OR “low rectal cancer”[Title/Abstract] OR “very low rectal cancer”[Title/Abstract] OR “ultra low rectal cancer”[Title/Abstract] OR “rectal carcinoma”[Title/Abstract] OR “neoplasm rectal”[Title/Abstract] OR “rectal neoplasm”[Title/Abstract] OR “neoplasms rectal”[Title/Abstract] OR “rectum neoplasms”[Title/Abstract] OR “neoplasm rectum”[Title/Abstract] OR “rectum neoplasm”[Title/Abstract] OR “rectal tumors”[Title/Abstract] OR “rectal tumor”[Title/Abstract] OR “tumor rectal”[Title/Abstract] OR “cancer of rectum”[Title/Abstract] OR “rectum cancers”[Title/Abstract] OR “cancer of the rectum”[Title/Abstract] OR “rectal cancer”[Title/Abstract] OR “cancer rectal”[Title/Abstract] OR “rectal cancers”[Title/Abstract] OR “rectum cancer”[Title/Abstract] OR “cancer rectum”[Title/Abstract]) AND (“neoadjuvant therapy”[Title/Abstract] OR “neoadjuvant chemoradiotherapy”[Title/Abstract] OR “neoadjuvant chemoradiation therapy”[Title/Abstract] OR “neoadjuvant chemoradiation treatment”[Title/Abstract] OR “neoadjuvant chemoradiation”[Title/Abstract] OR “neoadjuvant radiotherapy”[Title/Abstract] OR “neoadjuvant radiation treatment”[Title/Abstract] OR “neoadjuvant radiation therapy”[Title/Abstract] OR “neoadjuvant radiation”[Title/Abstract] OR “neoadjuvant chemotherapy”[Title/Abstract] OR “neoadjuvant chemotherapy treatment”[Title/Abstract] OR “neoadjuvant systemic therapy”[Title/Abstract] OR “preoperative chemoradiotherapy”[Title/Abstract]) AND (“immune checkpoint inhibitors”[Title/Abstract] OR “immune checkpoint blockers”[Title/Abstract] OR “pd l1 inhibitors”[Title/Abstract] OR “programmed death ligand 1 inhibitors”[Title/Abstract] OR “pd 1 pd l1 blockade”[Title/Abstract] OR “pd 1 inhibitors”[Title/Abstract] OR ((“immune checkpoint inhibitors”[Pharmacological Action] OR “immune checkpoint inhibitors”[Supplementary Concept] OR “immune checkpoint inhibitors”[All Fields] OR “programmed cell death protein 1 inhibitor”[All Fields] OR “immune checkpoint inhibitors”[MeSH Terms] OR (“Immune”[All Fields] AND “Checkpoint”[All Fields] AND “Inhibitors”[All Fields])) AND “immune therapy”[Title/Abstract]) OR “immunization therapy”[Title/Abstract] OR “immunotherapeutic”[Title/Abstract] OR “immunotherapy”[Title/Abstract] OR “immunotherapy treatment”[Title/Abstract] OR “PD-1”[Title/Abstract] OR “PD-L1”[Title/Abstract] OR “CTLA-4”[Title/Abstract] OR “pembrolizumab”[Title/Abstract] OR “nivolumab”[Title/Abstract] OR “dostarlimab”[Title/Abstract] OR “sintilimab”[Title/Abstract] OR “toripalimab”[Title/Abstract] OR “ipilimumab”[Title/Abstract] OR “tremelimumab”[Title/Abstract] OR “atezolizumab”[Title/Abstract] OR “durvalumab”[Title/Abstract] OR “avelumab”[Title/Abstract] OR “camrelizumab”[Title/Abstract] OR “tislelizumab”[Title/Abstract] OR “cemiplimab”[Title/Abstract]) AND ((“sphincter sparing surgery”[Title/Abstract] OR “sphincter preservation”[Title/Abstract] OR “anal preservation”[Title/Abstract] OR “anal sphincter preservation”[Title/Abstract] OR “anterior resection”[Title/Abstract])))”. The language was restricted to Chinese and English. In addition, we evaluated the references of the included articles and selected more relevant studies.

### 2.2 Eligibility criteria and study selection

Eligibility criteria were: (1) Randomized Controlled Trials (RCTs), single-arm trials and observational studies (including cohort studies and case-control studies) involving patients diagnosed with rectal cancer; (2) studies focusing on the combination of neoadjuvant therapy and immunotherapy for rectal cancer patients (neoadjuvant therapy included but not limited to chemotherapy, radiotherapy, targeted therapy, and immunotherapy included immune checkpoint inhibitors, adoptive cellular immunotherapy, etc.); (3) studies reporting at least one of the following outcome indicators: sphincter preservation rate of rectal cancer patients, clinical efficacy indicators (including objective response rate, pathological complete response rate, disease control rate), or safety indicators (including incidence of adverse events, severity of adverse events, incidence of treatment-related adverse events).

Titles and abstracts of potential studies were independently screened by two authors. Irrelevant studies were excluded based on the following criteria: (1) duplicate studies from the same trials or duplicate publications of the same research data; (2) review articles, meta-analyses, case reports, letters to the editor, conference abstracts and other non-original research literature; (3) studies that did not involve the combination of neoadjuvant therapy and immunotherapy (e.g., single neoadjuvant therapy or single immunotherapy alone); (4) studies that did not report sphincter preservation rate, clinical efficacy or safety-related outcome indicators; (5) studies with incomplete data, unclear research design or inability to extract effective data after full-text review. Full texts were assessed for eligibility when abstracts provided insufficient information to determine whether the study met the inclusion criteria. Discrepancies between the two screeners during title/abstract screening and full-text assessment were resolved through discussion with the senior author. The Grading of Recommendations Assessment Development and Evaluation (GRADE) approach was applied to evaluate the certainty of cumulative evidence for all primary and secondary outcomes of interest, including sphincter preservation rate, R0 resection rate, pathological complete response rate, tumor regression grade, overall complete response rate, and adverse events. The sphincter preservation rate was the pre-specified primary outcome; R0 resection rate, pathological complete response rate, tumor regression grade, overall complete response rate, adverse events, and postoperative complications were pre-specified secondary outcomes. The initial certainty of evidence was rated as high for RCTs and low for single-arm studies, and then systematically downgraded or upgraded based on five key domains: risk of bias, inconsistency of results, imprecision of effect estimates, indirectness of evidence, and publication bias. Specifically, the evidence was downgraded by one or two levels for serious or very serious limitations in each domain, and upgraded only when there was a large magnitude of treatment effect, dose-response relationship, or adequate control of confounding factors in observational studies. The certainty of evidence was ultimately categorized into four levels: high, moderate, low, and very low. Two authors independently conducted the GRADE assessment, with the kappa coefficient used to test the inter-rater consistency. Any discrepancies in the assessment results were resolved through discussion and consensus with a senior author, and a Summary of Findings table was constructed to present the GRADE assessment results and key clinical outcome data in a concise and standardized manner.

### 2.3 Data extraction and evaluation of the risk of bias

The characteristics of included studies were extracted, including study, year, trial type (RCT/single-arm study), Study site (nation, multi/singe center), age (years), sex(male/female), pathological stage, etc. Extracted outcomes included sphincter preservation rate, R0 resection rate, pathological complete response rate, tumor regression grade, overall complete response rate, and adverse events. To ensure consistency across studies, the outcomes were defined *a priori* and the denominator used for pooling each proportion is specified below. The sphincter preservation rate was defined as the proportion of patients who successfully retained the anal sphincter without permanent colostomy among all rectal cancer patients who underwent curative surgery in the included studies. The overall complete response rate was defined as the proportion of patients achieving either clinical complete response or pathological complete response among the intention-to-treat population who received neoadjuvant therapy and completed response assessment. The pathological complete response rate was defined as the proportion of patients with no viable tumor cells in the resected primary lesion and all examined lymph nodes among those who underwent surgery and had a complete pathological examination. Tumor regression grade (TRG) was assessed according to the grading system used in each study (e.g., AJCC/CAP, Mandard, or Dworak). The TRG 0–1 rate was defined as the proportion of patients with complete or near-complete tumor regression (the best two response categories) among those who underwent surgery and had evaluable TRG. The TRG ≥2 rate was defined as the proportion of patients with a regression grade of 2 or higher (indicating a less favorable response) in the same population. The R0 resection rate was defined as the proportion of patients with microscopically negative resection margins among all patients who underwent curative surgery and had their margin status assessed. The postoperative complications rate was defined as the proportion of patients who developed at least one postoperative complication within a specified period after surgery among all surgical patients. Adverse events were graded according to the Common Terminology Criteria for Adverse Events (CTCAE). The rate of grade 1–2 adverse events was defined as the proportion of patients experiencing at least one grade 1 or 2 event, and the rate of grade ≥3 adverse events was defined as the proportion experiencing at least one grade 3 or higher event. Both adverse event rates were calculated in the safety analysis set, which included all patients who received at least one dose of the study treatment and underwent safety evaluation.Two authors independently assessed the quality using the Risk Of Bias In Non - randomized Studies - of Interventions(ROBINS-I) tool, the Cochrane Collaboration risk of bias tool, and the GRADE assessment. Consistency was assessed using the kappa coefficient. Any disagreements were resolved through consensus with the senior author.

### 2.4 Statistical analysis

All data in this meta-analysis were analyzed with R Studio 4.3.2 software (R Foundation for Statistical Computing, Vienna, Austria). For baseline characteristics presented as median, interquartile range (IQR) and range, values were converted to mean ± standard deviation using established mathematical formulas for descriptive synthesis.

The primary analysis of all rate-based efficacy and safety outcomes was conducted using proportion meta-analysis, with detailed statistical specifications as follows. For data transformation, the Freeman-Tukey double arcsine transformation was applied to the raw proportion of each included study prior to pooling. This method stabilizes the variance of proportion estimates and improves the approximation to a normal distribution, particularly for extreme values near 0 or 1. After pooled calculation, all summary estimates were back-transformed to the original proportion scale for final result reporting.

For effect model estimation, between-study heterogeneity was evaluated via the chi-squared test and quantified using the I² statistic. A P-value < 0.1 for the chi-squared test or an I² value > 50% was considered to indicate statistically significant heterogeneity. When significant heterogeneity was present, a random-effects model was adopted, with the between-study variance (τ²) estimated by the DerSimonian-Laird method to derive the pooled proportion and its 95% confidence interval. When heterogeneity was low (I² < 50% and P > 0.1), a common-effect (fixed-effect) model based on inverse-variance weighting of the transformed proportions was applied. A P-value < 0.05 for the pooled estimate was defined as statistically significant.

For handling of zero-event studies, benefiting from the Freeman-Tukey double arcsine transformation, studies with zero events or 100% event rates could be directly included in the meta-analysis without additional continuity correction (e.g., adding 0.5 to all cells of the contingency table). For any outcome where all included studies reported zero events, only qualitative description was conducted, and quantitative pooling was not performed.

In addition, sensitivity analysis was performed to evaluate the stability and reliability of the pooled results.

## 3 Results

### 3.1 Study selection

**Figure 1:**
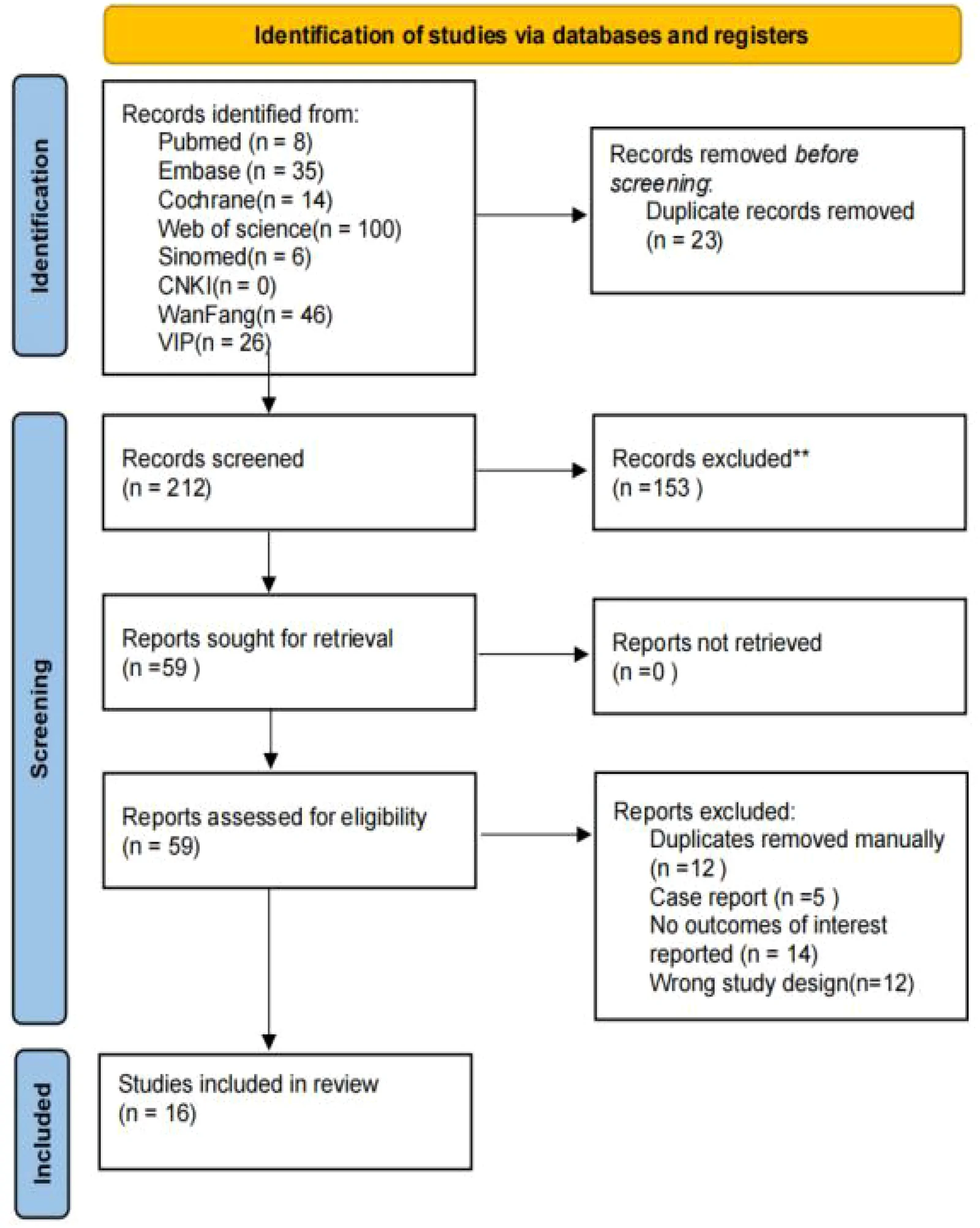
Flow diagram of meta-analysis for inclusion/exclusion of studies.

The initial search yielded a total of 235 published relevant studies from eight databases (PubMed = 8, Embase = 35, Cochrane Library = 14, Web of Science = 100, Sinomed = 6, CNKI = 0, WanFang = 46 and VIP = 26). About twenty-three studies were retained after removing duplicates and screening the titles and abstracts. Then, the remaining full-text articles were carefully assessed. Finally, sixteen studies met the inclusion criteria and were included in this meta-analysis. The flowchart of the selection process is shown in **Figure 1**. The details of each included study are described in **Table 1**.

**Table 1:** Characteristics of the studies included in the meta-analysis.

No	Study,year	Trial (RCT/Single-arm study)	Study site (Nation, Multi/Single center)	Age (years)	Sex(Male/Female)	Pathological Stage	Long Course/Short Course	Combination/Sequential therapy	Tumor anal verge distance	Tumor longest diameter	MRF positivity rate	EMVI positivity rate	Neoadjuvant to surgery interval (weeks)	Immunohistochemistry
1	Jiale Gao,2022 (15)	Single- arm study	China,Single center	57.1 ± 13.1	9/2	II/III=5/6	Long Course	Combination	5.1 ± 2.1cm	NDA	NDA	NDA	6.7~12	pMMR
2	Yunlin Zhao,2021 (16)	Single- arm study	China,Single center	57 (31~73)	7/9	II/III=3/13	Short Course	Sequential	6.1(3~10)cm	5.2(2.1-10)cm	NDA	NDA	8 (4~12)	pMMR/dMMR/unknown=8/2/6
3	Xinliang Liu, 2024 (17)	Single- arm study	China,Single center	61 (34~74)	27/12	II/III=5/34	Long Course	Combination	4(0~9)cm	NDA	24/39	21/39	17 (10~27)	NDA
4	Leqi Zhou, 2022 (18)	Single- arm study	China,Single center	55 (39~71)	12/6	I/II/III=8/5/5	Long Course	Combination	1(0~2)cm	NDA	NDA	NDA	NDA	pMMR
5	Peng Zhang, 2022 (19)	Single- arm study	China,Single center	57 ± 16	17/13	II/III=4/26	Short Course	Sequential	4.7(1.9~9)cm	5.4(2.1~10)cm	NDA	12/26	14 (5~141)	pMMR/dMMR/unknown=28/1/1
6	Qiya Wang,2023 (20)	RCT	China,Single center	53 (27~69)	78/46	II/III=2/32	Short Course	Sequential	NDA	NDA	17/62	11/62	NDA	pMMR/dMMR=59/3
7	Yingjie Li,2021 (21)	Single- arm study	China,Single center	65 (47~78)	15/9	NDA	Long Course	Sequential	4(3~7)cm	5.1(2.1-7.5) cm	10/24	10/24	NDA	pMMR
8	Leqi Zhou,2023 (22)	Single- arm study	China,multicenter	55 (38~75)	14/9	NDA	Long Course	Sequential	NDA	NDA	NDA	NDA	NDA	NDA
9	Y. Wang,2023 (23)	RCT	China,multicenter	55	NDA	III=91	Short Course	Combination	≤10cm	NDA	86/104	86/104	NDA	NDA
10	Y. Takahashi,2023 (24)	Single- arm study	Japan,Single center	63 (24~75)	18/7	II/III=13/12	Long Course	Sequential	≤12cm	NDA	NDA	NDA	NDA	PMMR/dMMR=24/1
11	Jianhong Peng,2025 (25)	Single- arm study	China,Single center	59 (54~66)	11/11	NDA	Long Course	Sequential	4(3-5)cm	NDA	NDA	NDA	NDA	NDA
12	Jiarui Lin,2025 (26)	Single- arm study	China,Single center	64 (33~78)	NDA	NDA	Long Course	Sequential	4.1(1.5~9)cm	NDA	21/29	12/29	NDA	NDA
13	BoFeng,2025 (27)	Single- arm study	China,Single center	NDA	NDA	NDA	Short Course	Sequential	NDA	NDA	NDA	NDA	8	PMMR
14	Y. Chen,2024 (28)	Single- arm study	China,Single center	NDA	12/11	NDA	Short Course	Sequential	3.8(2.2-11.9) cm	NDA	15/23	17/23	2~4	pMMR
15	Zhuo Chen,2024 (29)	RCT	China,Single center	52.00(48.75,59.00)	15/5	NDA	Long Course	Sequential	5(4~6.5)cm	5.6(5.1~6.08) cm	NDA	20/20	NDA	NDA
				55.50(49.50,66.25)	6/10	NDA	Long Course	Sequential	5(2~6)cm	5(4~6.5)cm	NDA	16/16	NDA	NDA
				51.00(39.00,59.25)	8/6	NDA	Short Course	Sequential	5(2~6)cm	5(4~6.5)cm	NDA	14/14	NDA	NDA
				56.00(51.00,60.75)	9/3	NDA	Short Course	Sequential	5.25(4.28~6.32)cm	5.3(4~9.48) cm	NDA	12/12	NDA	NDA
16	Thomas J George,2022 (30)	Single- arm study	America,Single center	NDA	NDA	NDA	NDA	Sequential	NDA	NDA	NDA	NDA	8~12	NDA

### 3.2 Risk of bias assessment

ROBINS-I (31) is a new tool used for evaluating the risk of bias in observational studies. Cochrane Collaboration risk of bias tool was utilized for assessing the risk of bias in randomized controlled trials. Detailed information can be found in **Table 2**. Among 13 single-arm interventional study, 6 (46.2%) were judged to be high risk (mainly due to serious confounding and follow-up bias), 7 as moderate risk, and none as low risk. The high bias risk in these 6 studies was attributed to inherent limitations of observational designs rather than individual methodological flaws: insufficient adjustment for potential confounders (e.g.,age,comorbidities) and incomplete/inconsistent follow-up in a few studies, common challenges in non-randomized research.

**Table 2:** Risk of bias assessment.

Study	Type of bias							
	Bias due to confounding	Bias due to selection of participants	Bias due to exposure classification	Bias due to misclassification during follow-up	Bias due to missing data	Bias due to measurement of the outcome	Bias due to selective reporting of the results	Overall rating
Jiale Gao, 2022 (15)	Low	Low	Low	Low	Low	Moderate	Low	Moderate
Yunlin Zhao, 2021 (16)	Low	Moderate	Low	Low	Low	Moderate	Low	Moderate
Xinliang Liu, 2024 (17)	Serious	Low	Low	Serious	Low	Moderate	Low	High
Leqi Zhou, 2022 (18)	Low	Low	Low	Low	Low	Moderate	Low	Moderate
Peng Zhang, 2022 (19)	Serious	Moderate	Low	Serious	Low	Moderate	Low	High
Yingjie Li,2021 (21)	Low	Moderate	Low	Low	Low	Moderate	Low	Moderate
Leqi Zhou,2023 (22)	Serious	Serious	Low	Low	Low	Moderate	Low	High
Y. Takahashi,2023 (24)	Low	Low	Low	Low	Low	Moderate	Low	Moderate
Jianhong Peng,2025 (25)	Moderate	Low	Low	Moderate	Low	Moderate	Low	Moderate
Jiarui Lin,2025 (26)	Serious	Low	Low	Serious	Low	Moderate	Low	High
BoFeng,2025 (27)	Low	Moderate	Low	Low	Low	Moderate	Low	Moderate
Y. Chen,2024 (28)	Serious	Moderate	Low	Serious	Low	Moderate	Low	High
Thomas J George,2022 (30)	Serious	Serious	Low	Low	Low	Moderate	Low	High
Kappa	0.72	0.9	1	1	1	1	1	0.85
Study	Type of bias							
	Random sequence generation	Allocation concealment	Blinding of participants and personnel	Blinding of outcome assessment	Incomplete outcome data addressed	Selective reporting		Overall rating
Qiya Wang,2023 (20)	Low	Low	Low	Low	Low	Low		Low
Y. Wang,2023 (23)	Low	Low	Low	Low	Low	Low		Low
Zhuo Chen,2024 (29)	Low	Low	Low	Low	Low	Low		Low
Kappa	1	1	1	1	1	1		1

To minimize bias impact, we used standardized tools for objective assessment. Subgroup analysis confirmed that excluding high-risk studies did not alter the main result direction. Additionally, all 3 RCTs were low risk across critical domains, providing high-quality evidence to support result robustness.

Inter-rater agreement was substantial to almost perfect (Kappa: 0.72–1.00), confirming assessment reliability. Subgroup analysis excluding high-risk studies showed consistent direction of effect, which partially supports the robustness of the overall findings; however, the overall certainty of evidence remains limited by the high proportion of non-randomized studies.

### 3.3 The grading of recommendations assessment

The certainty of cumulative evidence for all efficacy and safety outcomes of neoadjuvant therapy in rectal cancer was evaluated using the GRADE approach (32), and the results were summarized in the **Table 3**. For core outcomes, the certainty of evidence for sphincter preservation rate (16 studies, n=540) was rated as moderate, with moderate risk of bias (I²=37%) and no serious imprecision of effect estimates. In contrast, the remaining core outcomes, including R0 resection rate (9 studies, n=206), grade 1–2 adverse event (AE) rate (3 studies, n=79) and grade ≥3 AE rate (7 studies, n=185), were all rated as low certainty of evidence, mainly due to high risk of bias and/or substantial between-study heterogeneity (I²=51% for R0 resection rate, I²=97% for grade ≥3 AE rate).

**Table 3:** Summary of findings for GRADE assessment of neoadjuvant therapy in rectal cancer.

Outcome level	Outcome	N studies	Total sample	Pooled Proportion	CI 95	Heterogeneity I^2^	Risk of bias	Final GRADE quality
Core	Sphincter preservation rate	16	540	0.90	0.87-0.93	37%	Moderate	Moderate
Core	R0 resection rate	9	206	0.98	0.96-1	51%	High	Low
Important	pCR rate	16	443	0.34	0.29-0.38	72%	Moderate	Low
Important	TRG 0–1 rate	8	184	0.55	0.49-0.62	79%	High	Low
Important	TRG ≥2 rate	5	96	0.28	0.2-0.37	0%	High	Low
Important	Overall CR rate	12	343	0.49	0.44-0.54	66%	Moderate	Low
Core	AE Grade 1–2 rate	3	79	0.80	0.71-0.89	0%	High	Low
Core	AE Grade ≥3 rate	7	185	0.17	0.13-0.21	97%	High	Low
Important	Postoperative complications rate	5	125	0.24	0.18-0.3	92%	High	Low

For all important secondary outcomes, including pCR rate (16 studies, n=443), tumor regression grade (TRG) 0–1 rate (8 studies, n=184), TRG ≥2 rate (5 studies, n=96), overall complete response (CR) rate (12 studies, n=343) and postoperative complications rate (5 studies, n=125), the certainty of evidence was uniformly rated as low. The main downgrading factors were moderate/high risk of bias and significant heterogeneity across included studies (I² range: 0%-92%), with the highest heterogeneity observed for postoperative complications rate (I²=92%) and grade ≥3 AE rate (I²=97%). No upgrading factors were identified for all outcomes in this study, and no serious indirectness or publication bias was found.

### 3.4 Efficacy assessment

**Figure 2:**
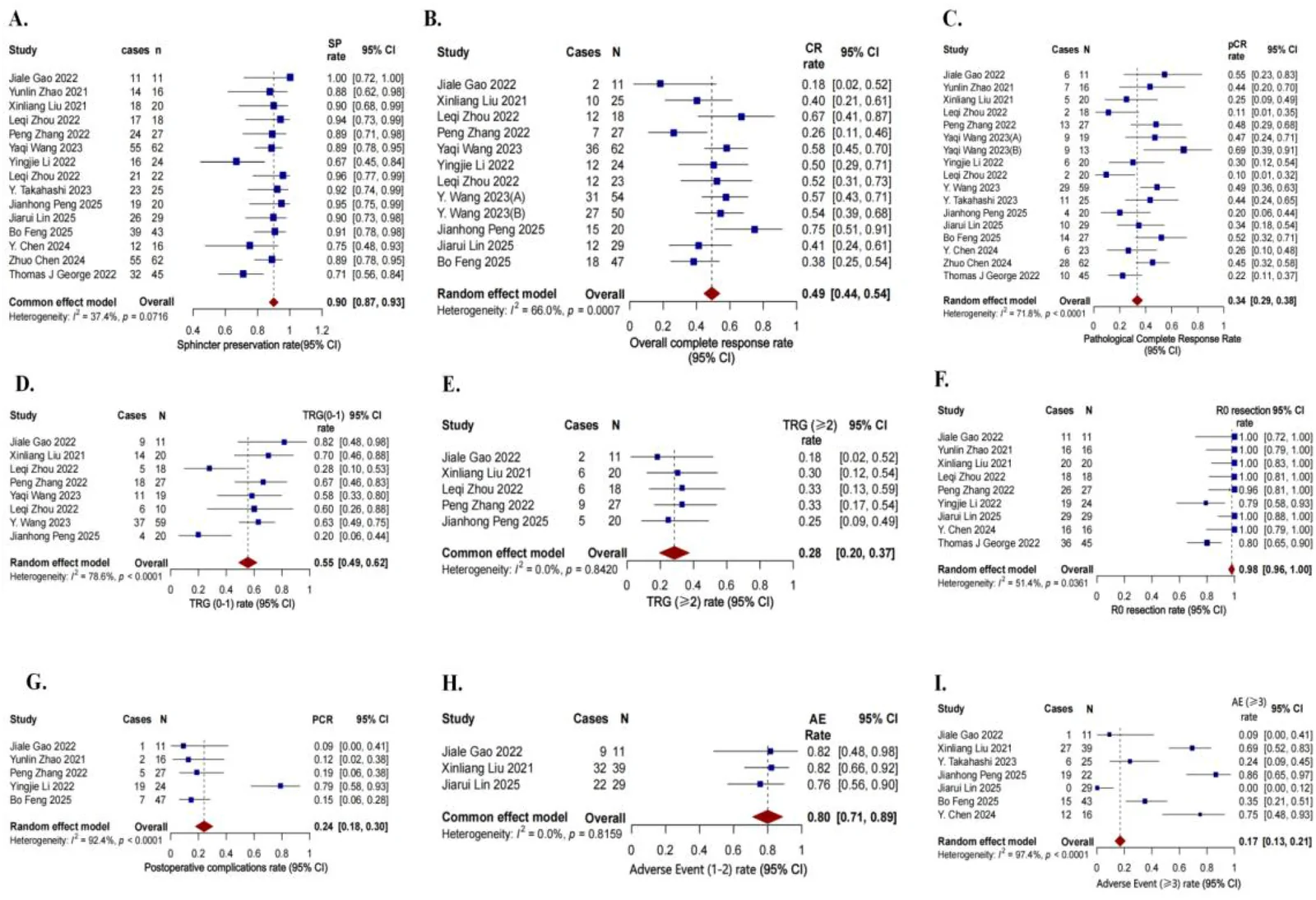
Forest plot of efficacy and safety outcomes in rectal cancer patients (**(A)** Sphincter preservation rate. **(B)** Overall complete response rate. **(C)** Pathological complete response rate. **(D)** Tumor regression grade 0–1 rate. **(E)** Tumor regression grade ≥2 rate. **(F)** R0 resection rate. **(G)** Postoperative complications rate. **(H)** Adverse events rate. **(I)** Adverse events (grade ≥3) rate).

**Figure 3:**
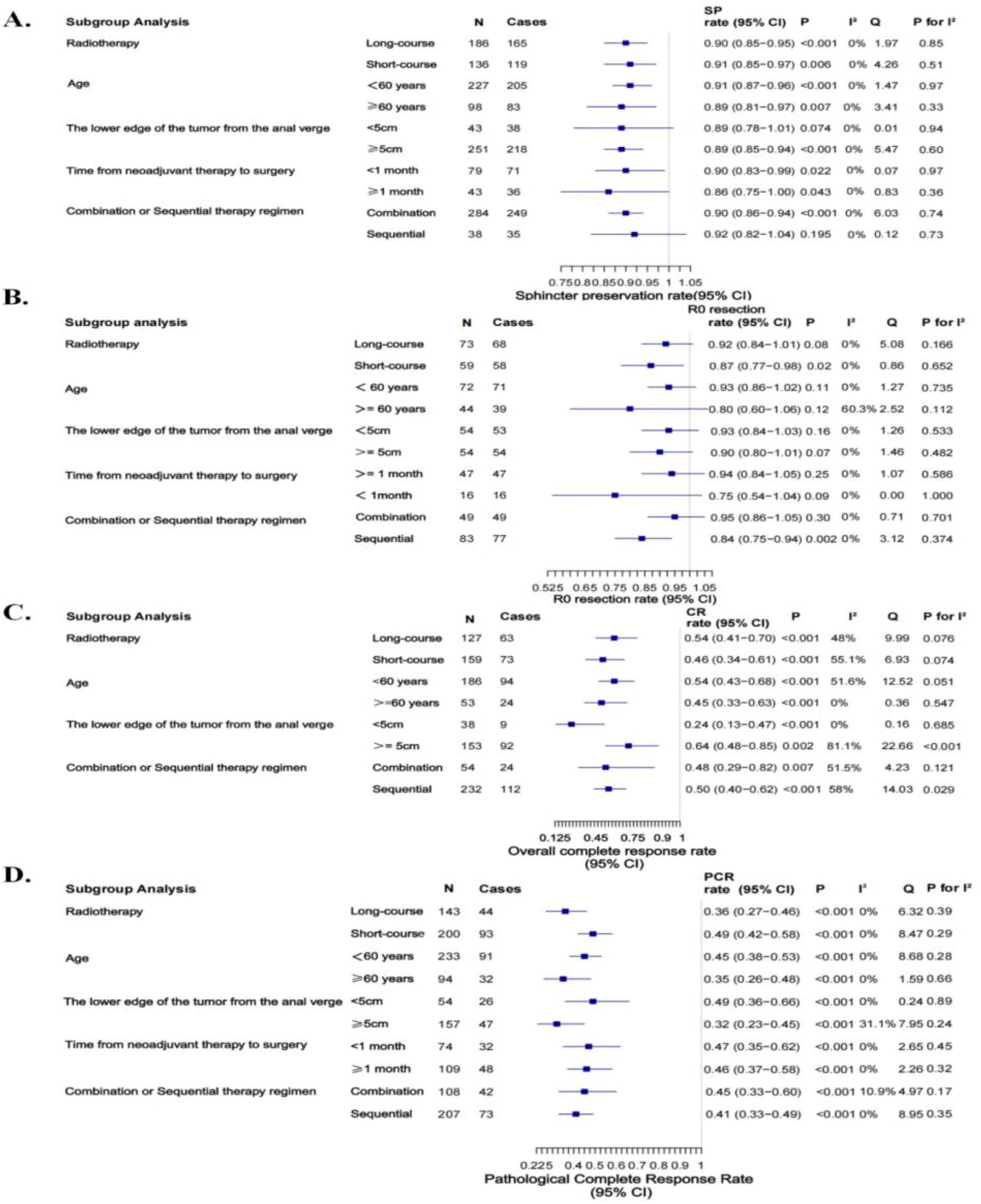
Subgroup analysis forest plot of efficacy outcomes in rectal cancer patients. (**(A)** Subgroup analysis of sphincter preservation rate. **(B)** Subgroup analysis of R0 resection rate. **(C)** Subgroup analysis of overall complete response rate. **(D)** Subgroup analysis of pathological complete response rate).

Sixteen studies (Jiale Gao et al., 2022 (15) ; Yunlin Zhao et al., 2021 (16); Xinliang Liu et al., 2024 (17); Leqi Zhou et al., 2022 (18); Peng Zhang et al., 2022 (19); Qiya Wang et al., 2023 (20); Yingjie Li et al., 2021 (21); Leqi Zhou et al., 2023 (22); Y. Wang et al., 2023 (23); Y. Takahashi et al., 2023 (24); Jianhong Peng et al., 2025 (25); Jiarui Lin et al., 2025 (26); Bo Feng et al., 2025 (27); Y. Chen et al., 2024 (28); Zhuo Chen et al., 2024 (29); Thomas J George et al., 2022 (30)) have reported efficacy for sphincter-preserving rate in which a total of 540 patients were enrolled. The corresponding forest plot and subgroup analysis results are presented in **Figures 2**, **3** respectively.

#### 3.4.1 Sphincter preserving rate (primary outcome)

A total of 16 studies involving 540 patients were included in this meta-analysis. The overall sphincter preservation rate was estimated using a random−effects model as 0.90 (95%CI: 0.87–0.93).Low-to-moderate between−study heterogeneity was observed (I²=37.4%), with a borderline statistically significant heterogeneity test (*P* = 0.0716).The pooled estimate suggested a high overall sphincter preservation rate, though the precision and generalizability of the estimate are limited by the predominance of single-arm studies. The heterogeneity test met the pre-specified threshold for significance (*P* < 0.1), and together with the moderate I² value this indicated a potential source of variability across included studies, which warrants consideration when interpreting the pooled effect in the context of clinical protocols and patient populations. All *P* values reported within subgroups are from one-sample tests for the pooled proportion within each subgroup (testing whether the rate differs from zero), and should not be interpreted as between-subgroup comparisons.

Across all subgroups, pooled sphincter preservation rates were consistently high, with negligible within-subgroup heterogeneity. These are pooled estimates derived independently within each subgroup, not formal statistical comparisons between subgroups. By radiotherapy regimen, the pooled rate was 0.90 (95% CI: 0.85-0.95, *P* < 0.001) for long-course and 0.91 (95% CI: 0.85-0.97, *P* = 0.006) for short-course radiotherapy. By age, it was 0.91 (95% CI: 0.87-0.96, *P* < 0.001) for patients <60 years and 0.89 (95% CI: 0.81-0.97, *P* = 0.007) for those≥60 years. By distance from the tumor’s lower edge to the anal verge, it was 0.89 (95% CI: 0.78-1.01, *P* = 0.074) for tumors <5 cm and 0.89 (95% CI: 0.85-0.94, *P* < 0.001) for tumors ≥5 cm. By interval from neoadjuvant therapy to surgery, it was 0.90 (95% CI: 0.83-0.99, *P* = 0.022) for <1 month and 0.86 (95% CI: 0.75-1.00, *P* = 0.043) for ≥1 month. By treatment modality, it was 0.90 (95% CI: 0.86-0.94, *P* < 0.001) for combination therapy and 0.92 (95% CI: 0.82-1.04, *P* = 0.195) for sequential therapy.The Forest plot of overall sphincter preservation rate has been shown in **Figure 2A**. The Subgroup analysis of sphincter preservation rate has been shown in **Figure 3A**.

#### 3.4.2 R0 resection rate (secondary outcome)

A total of 9 studies involving 206 patients were included in this meta-analysis of R0 resection rate. Using a random-effects model, the overall R0 resection rate was 0.98 (95% CI: 0.96–1.00), with moderate between-study heterogeneity (I²=51.4%, *P* = 0.0361).

Across most subgroups, pooled R0 resection rates showed negligible within-subgroup heterogeneity. The exception was the ≥60 years age subgroup, which showed moderate heterogeneity (I²= 60.3%), though this did not reach statistical significance (*P* for I²= 0.112). These are pooled estimates derived independently within each subgroup, not formal statistical comparisons between subgroups.

By radiotherapy regimen, the pooled R0 resection rate was 0.92 (95% CI: 0.84-1.01, *P* = 0.08) for long-course and 0.87 (95% CI: 0.77-0.98, *P* = 0.02) for short-course radiotherapy. By age, it was 0.93 (95% CI: 0.86-1.02, *P* = 0.11) for patients <60 years and 0.80 (95% CI: 0.60-1.06, *P* = 0.12) for those ≥60 years. By distance from the tumor’s lower edge to the anal verge, it was 0.93 (95% CI: 0.84-1.03, *P* = 0.16) for tumors <5 cm and 0.90 (95% CI: 0.80-1.01, *P* = 0.07) for tumors ≥5 cm. By interval from neoadjuvant therapy to surgery, it was 0.94 (95% CI: 0.84-1.05, *P* = 0.25) for ≥1 month and 0.75 (95% CI: 0.54-1.04, *P* = 0.09) for <1 month. By treatment modality, it was 0.95 (95% CI: 0.86-1.05, *P* = 0.3) for combination therapy and 0.84 (95% CI: 0.74–0.94, *P* = 0.002) for sequential therapy.The Forest plot of overall R0 resection rate has been shown in **Figure 2F**. The Subgroup analysis of R0 resection rate has been shown in **Figure 3B**.

#### 3.4.3 Pathological complete response rate (secondary outcome)

A total of 16 studies involving 443 patients were included in this meta-analysis of pathological complete response (pCR) rate. Using a random-effects model, the overall pCR rate was 0.34 (95% CI: 0.29–0.38), with substantial between-study heterogeneity (I²=71.8%, *P* < 0.0001).

Across most subgroups, pooled pCR rates showed negligible within-subgroup heterogeneity. Two subgroups showed non-zero heterogeneity—tumors ≥5 cm from the anal verge (I²= 31%) and the combination therapy group (I²= 11%), though neither reached statistical significance (*P* for I²= 0.24 and 0.17, respectively). These are pooled estimates derived independently within each subgroup, not formal statistical comparisons between subgroups.

By radiotherapy regimen, the pooled pCR rate was 0.36 (95% CI: 0.27-0.46, *P* < 0.001) for long-course and 0.49 (95% CI: 0.42-0.58, *P* < 0.001) for short-course radiotherapy. By age, it was 0.45 (95% CI: 0.38-0.53, *P* < 0.001) for patients <60 years and 0.35 (95% CI: 0.26–0.48, *P* < 0.001) for those ≥60 years. By distance from the tumor’s lower edge to the anal verge, it was 0.49 (95% CI: 0.36–0.66, *P* < 0.001) for tumors <5 cm and 0.32 (95% CI: 0.23-0.45, *P* < 0.001) for tumors ≥5 cm. By interval from neoadjuvant therapy to surgery, it was 0.47 (95% CI: 0.35-0.62, *P* < 0.001) for <1 month and 0.46 (95% CI: 0.37-0.58, *P* < 0.001) for ≥1 month. By treatment modality, it was 0.45 (95% CI: 0.33-0.60, *P* < 0.001) for combination therapy and 0.41 (95% CI: 0.33-0.49, P < 0.001) for sequential therapy.The Forest plot of overall Pathological Complete Response Rate has been shown in **Figure 2C**. The Subgroup analysis of Pathological Complete Response Rate has been shown in **Figure 3D**.

#### 3.4.4 Tumor regression grade(0-1) rate (secondary outcome)

A total of 8 studies involving 184 patients were included in this meta-analysis of tumor regression grade (TRG 0-1) rate. Using a random-effects model, the overall TRG (0-1) rate was 0.55 (95% CI: 0.49–0.62), with substantial between-study heterogeneity (I²=78.6%, *P* < 0.0001).The Forest plot of Tumor Regression Grade(0-1) Rate has been shown in **Figure 2D**.

#### 3.4.5 Tumor regression grade(≥2) rate (secondary outcome)

A total of 5 studies involving 96 patients were included in this meta-analysis of tumor regression grade (TRG ≥2) rate. Using a common-effect model, the overall TRG (≥2) rate was 0.28 (95% CI: 0.20–0.37), with no between-study heterogeneity (I² = 0.0%, *P* = 0.842). The Forest plot of overall TRG(≥2) rate has been shown in **Figure 2E**.

#### 3.4.6 Overall complete response rate (secondary outcome)

A total of 12 studies involving 343 patients were included in this meta-analysis of overall complete response rate. Using a random-effects model, the overall CR rate was 0.49 (95% CI: 0.44–0.54), with substantial between-study heterogeneity (I²=66.0%, *P* = 0.0007).

Heterogeneity in the overall CR subgroups was more variable than in the other outcomes, with statistically significant heterogeneity observed only in the ≥5 cm subgroup (*P* for I²< 0.001). These are pooled estimates derived independently within each subgroup, not formal statistical comparisons between subgroups.

By radiotherapy regimen, the pooled overall CR rate was 0.54 (95% CI: 0.41-0.70, *P* < 0.001) for long-course, with moderate heterogeneity (I²= 48%, *P* for I²= 0.076), and 0.46 (95% CI: 0.34–0.61, *P* < 0.001) for short-course, with moderate heterogeneity (I²= 55.1%, *P* for I²= 0.074). By age, it was 0.54 (95% CI: 0.43-0.68, *P* < 0.001) for patients <60 years, with moderate heterogeneity approaching significance (I²= 51.6%, *P* for I²= 0.051), and 0.45 (95% CI: 0.33-0.63, *P* < 0.001) for those ≥60 years, with no heterogeneity (I²= 0%, *P* for I²= 0.547). By distance from the tumor’s lower edge to the anal verge, it was 0.24 (95% CI: 0.13-0.47, *P* < 0.001) for tumors <5 cm, with no heterogeneity (I²=0%, *P* for I²=0.685), and 0.64 (95% CI: 0.48-0.85, *P* = 0.002) for tumors≥5 cm, with substantial and statistically significant heterogeneity (I²=81.1%, *P* for I²< 0.001). By treatment modality, it was 0.48 (95% CI: 0.29-0.82, *P* = 0.007) for combination therapy, with moderate heterogeneity (I²=51.5%, *P* for I²=0.121), and 0.50 (95% CI: 0.40-0.62, *P* < 0.001) for sequential therapy, with moderate heterogeneity (I²=58%, *P* for I²=0.029).The Forest plot of overall CR rate has been shown in **Figure 2B**. The Subgroup analysis of overall CR rate has been shown in **Figure 3C**.

#### 3.4.7 Postoperative complications rate (secondary outcome)

A total of 5 studies involving 125 patients were included in this meta-analysis of postoperative complications rate. Using a random-effects model, the overall postoperative complications rate was 0.24 (95% CI: 0.18–0.30), with substantial between-study heterogeneity (I²=92.4%, *P* < 0.0001).The Forest plot of Postoperative complications rate has been shown in **Figure 2G**.

#### 3.4.8 Adverse event (secondary outcome)

##### 3.4.8.1 Adverse Event(1-2) rate

A total of 3 studies involving 79 patients were included in this meta-analysis of adverse event (AE, grade 1-2) rate. Using a common-effect model, the overall AE (grade 1-2) rate was 0.80 (95% CI: 0.71–0.89), with no between-study heterogeneity (I² = 0.0%, *P* = 0.8159).The Forest plot of Adverse Event(1-2) rate has been shown in **Figure 2H**.

##### 3.4.8.2 Adverse Event(≥3) rate

A total of 7 studies involving 185 patients were included in this meta-analysis of adverse event (AE, grade ≥3) rate. Using a random-effects model, the overall AE (grade ≥3) rate was 0.17 (95% CI: 0.13–0.21), with substantial between-study heterogeneity (I²=97.4%, *P* < 0.0001).The Forest plot of Adverse Event(grade ≥3) rate has been shown in **Figure 2I**.

## 4 Discussion

To date, no standardized optimal neoadjuvant regimen incorporating immunotherapy has been established for rectal cancer,and its optimal application model remains undetermined (33, 34). Several clinical studies have explored different combinations: for MSI-H/dMMR tumors, a phase 1b study (NCT05890742) showed ipilimumab N01 plus sintilimab achieved a 78.4% pCR rate (vs 46.7% for PD-1 monotherapy), with 100% R0 resection, no recurrence over 21.4-month median follow-up, and 71.2% TRAEs (3.8% grade ≥3) (35). For pMMR/MSS subtype, the STELLAR II study reported short-course radiotherapy plus chemotherapy and sintilimab increased clinical CR rate from 25.0% to 45.5% and pCR rate from 20.7% to 43.1%, with manageable grade 3–4 hematological toxicities and 5.5% severe immune-related adverse events (36). A domestic multicenter real-world study reported a 41.5% pCR rate among 94 patients (37).

Given inconsistencies among these single-center/single-arm studies, this meta-analysis synthesized data from 16 studies (540 patients), focusing on sphincter-preservation rate, surgical/pathological response, and adverse events.For conventional neoadjuvant therapy without immunotherapy in locally advanced rectal cancer, pooled evidence has demonstrated ranges of 72%–81% for sphincter-preservation rate, 94%–99% for R0 resection rate, and 12%–28% for pCR rate. In contrast, the combined immunotherapy-containing neoadjuvant therapy achieved a 0.90 sphincter-preservation rate (95% CI: 0.87–0.93), 0.98 R0 resection rate (95% CI: 0.96–1.00), and 0.34 pCR rate (95% CI: 0.29–0.38), of which the sphincter-preservation and pCR rates appear numerically higher than previously reported pooled estimates for conventional neoadjuvant chemoradiotherapy, whereas the R0 resection rate is comparable (38). However, this cross-study comparison should be interpreted with caution, as it is based on indirect evidence rather than head-to-head trials.

In exploratory descriptive subgroup analyses, several numerical differences were observed. The pooled pCR rate appeared higher in the short-course radiotherapy subgroup (0.49) than in the long-course subgroup (0.36), in patients aged <60 years (0.45) than in those ≥60 years (0.35), and in tumors located <5 cm from the anal verge (0.49) than in those ≥5 cm (0.32). These numerical differences might be partly explained by the differential immunomodulatory effects of radiotherapy schedules (41), potentially more robust immune responses in younger patients (42), and the favorable tumor microenvironment of low-lying lesions (43). The pooled sphincter-preservation rate was comparable across radiotherapy regimens (0.90 vs 0.91) and age groups (0.91 vs 0.89), which aligns with previous findings (39), and the high R0 resection rate was consistent with international reports (40).

However, because all subgroup estimates were derived from independent single-arm studies and no formal between-subgroup interaction tests were performed, these numerical differences should be interpreted as purely descriptive and hypothesis-generating, and require confirmation in future head-to-head trials.

Preoperative evaluation showed a 0.55 TRG 0–1 rate and 0.49 overall CR rate. Long-course radiotherapy (0.54 vs 0.46) and age <60 years (0.54 vs 0.45) were associated with higher CR rates. Notably, tumors ≥5 cm had a numerically higher pooled cCR rate than those <5 cm (0.64 vs 0.24). This discordance between cCR and pCR in low-lying tumors likely reflects the inherent limitations of clinical assessment (digital rectal examination and MRI) in the distal rectum, where post-radiation fibrosis and residual tumor are difficult to differentiate. Consequently, clinicians may under-report cCR in ultra-low lesions, rather than this representing true biological resistance (44). No significant differences in CR/pCR rates were found between concurrent and sequential regimens (45), offering clinical flexibility.

The combined therapy showed a generally tolerable but highly heterogeneous safety profile: grade 1–2 adverse events occurred in 0.80 (95% CI: 0.71–0.89) of patients, grade ≥3 in 0.17 (95% CI: 0.13–0.21), and postoperative complications in 0.24 (95% CI: 0.18–0.30). However, the pooled estimates for grade ≥3 adverse events and postoperative complications should be interpreted with considerable caution, because between-study heterogeneity was very high (I² = 97% and 92%, respectively), most likely reflecting differences in treatment regimens, adverse-event grading systems, and surgical factors across studies. In particular, the present pooled safety outcomes combined immune-related adverse events with surgery-related complications; because these differ in mechanism, time-course, and management, future studies—and ideally a re-analysis of these data—should report immune-related adverse events (irAEs) separately from surgical complications. Main grade ≥3 adverse events were gastrointestinal and hematological toxicities, consistent with known PD-1/PD-L1 inhibitor safety profiles (46).

This meta-analysis has several strengths: it specifically focuses on anal sphincter preservation outcomes, a clinically important endpoint that has not been systematically synthesized in prior reviews (n = 540), with standardized bias assessment (Kappa 0.72–1.00) and detailed subgroup analyses. Limitations include mostly single-arm/non-randomized studies (only 3 RCTs), majority in Chinese populations (limiting generalizability), incomplete clinical/pathological data, and lack of long-term survival outcomes. Moreover, because all pooled analyses—including the subgroup analyses—were based on study-level aggregate data rather than individual-patient data, the subgroup analyses are susceptible to ecological (aggregation) bias and can therefore be interpreted only as exploratory and hypothesis-generating. In addition, substantial statistical heterogeneity was present for several pooled outcomes (e.g., pCR, overall CR, TRG 0–1, grade ≥3 AEs, and postoperative complications, with I² values up to 97%), and the overall certainty of evidence (GRADE) was low to moderate across all outcomes. Accordingly, the pooled estimates demonstrated differing endpoint-specific certainty under the GRADE framework: sphincter preservation was rated as moderate certainty, whereas R0 resection, pCR, and grade≥3 adverse events were each rated as low certainty, allowing readers to directly link the strength of each signal to its corresponding level of certainty. Subgroup analyses should therefore be interpreted with caution.

Despite limitations, this study has potential clinical implications: neoadjuvant immunotherapy may be a promising option for rectal cancer, especially low rectal cancer requiring sphincter preservation. It should be emphasized, however, that these sphincter-preservation and pCR rates were derived predominantly from highly selected single-arm cohorts and may therefore not be generalizable to unselected rectal cancer populations; they are most directly relevant to well-selected patients with low rectal cancer or high-risk features who are being considered for organ-preservation strategies.

Future research directions include: 1) large-scale phase III RCTs comparing combined immunotherapy vs conventional chemoradiotherapy (47); 2) stratified studies by MMR/MSI/TMB to identify optimal candidates and biomarkers (48); 3) optimizing radiotherapy schedules, immunotherapeutic agents, and treatment sequences; and 4) long-term follow-up to evaluate survival and late adverse events (49).

In summary, this meta-analysis of predominantly single-arm studies suggests that neoadjuvant therapy combined with immunotherapy is associated with favorable pooled proportions of sphincter preservation (0.90), R0 resection (0.98), and pCR (0.34). The safety profile appears broadly manageable based on limited data, although the high heterogeneity across studies warrants caution. These findings support further investigation of this strategy, but confirmatory evidence from high-quality RCTs is required before clinical adoption.However, high-quality RCTs and long-term follow-up are needed for clinical validation and regimen optimization.

## 5 Conclusion

For patients with rectal cancer, the addition of immunotherapy to conventional neoadjuvant therapy suggests encouraging but preliminary pooled estimates of sphincter-preservation rate, R0 resection rate, and pCR rate, with a safety profile that appears broadly manageable based on limited data; however, the current evidence is of low-to-moderate certainty (GRADE) and is derived largely from non-randomized, single-arm studies, so these findings should be regarded as preliminary and require confirmation in adequately powered, high-quality randomized controlled trials.

## Data Availability

The original contributions presented in the study are included in the article/supplementary material. Further inquiries can be directed to the corresponding author.
